# The Direct Semi-Quantitative Detection of 18 Pathogens and Simultaneous Screening for Nine Resistance Genes in Clinical Urine Samples by a High-Throughput Multiplex Genetic Detection System

**DOI:** 10.3389/fcimb.2021.660461

**Published:** 2021-04-12

**Authors:** Zhaoyang Sun, Wenjian Liu, Jinghao Zhang, Su Wang, Feng Yang, Yi Fang, Wenrong Jiang, Li Ding, Hu Zhao, Yanmei Zhang

**Affiliations:** ^1^ Department of Laboratory Medicine, Huadong Hospital, Affiliated With Fudan University, Shanghai, China; ^2^ Key Laboratory of Clinical Geriatric Medicine, Shanghai, China; ^3^ Research Center on Aging and Medicine, Fudan University, Shanghai, China

**Keywords:** UTIs, UTI-HMGS, uropathogens, resistance genes, semi-quantitative detection

## Abstract

**Background:**

Urinary tract infections (UTIs) are one the most common infections. The rapid and accurate identification of uropathogens, and the determination of antimicrobial susceptibility, are essential aspects of the management of UTIs. However, existing detection methods are associated with certain limitations. In this study, a new urinary tract infection high-throughput multiplex genetic detection system (UTI-HMGS) was developed for the semi-quantitative detection of 18 pathogens and the simultaneously screening of nine resistance genes directly from the clinical urine sample within 4 hours.

**Methods:**

We designed and optimized a multiplex polymerase chain reaction (PCR) involving fluorescent dye-labeled specific primers to detect 18 pathogens and nine resistance genes. The specificity of the UTI-HMGS was tested using standard strains or plasmids for each gene target. The sensitivity of the UTI-HMGS assay was tested by the detection of serial tenfold dilutions of plasmids or simulated positive urine samples. We also collected clinical urine samples and used these to perform urine culture and antimicrobial susceptibility testing (AST). Finally, all urine samples were detected by UTI-HMGS and the results were compared with both urine culture and Sanger sequencing.

**Results:**

UTI-HMGS showed high levels of sensitivity and specificity for the detection of uropathogens when compared with culture and sequencing. In addition, ten species of bacteria and three species of fungi were detected semi-quantitatively to allow accurate discrimination of significant bacteriuria and candiduria. The sensitivity of the UTI-HMGS for the all the target genes could reach 50 copies per reaction. In total, 531 urine samples were collected and analyzed by UTI-HMGS, which exhibited high levels of sensitivity and specificity for the detection of uropathogens and resistance genes when compared with Sanger sequencing. The results from UTI-HMGS showed that the detection rates of 15 pathogens were significantly higher (P<0.05) than that of the culture method. In addition, there were 41(7.72%, 41/531) urine samples were positive for difficult-to-culture pathogens, which were missed detected by routine culture method.

**Conclusions:**

UTI-HMGS proved to be an efficient method for the direct semi-quantitative detection of 18 uropathogens and the simultaneously screening of nine antibiotic resistance genes in urine samples. The UTI-HMGS could represent an alternative method for the clinical detection and monitoring of antibiotic resistance.

## Introduction

Urinary tract infections (UTIs) remain one of the most common infections among both outpatients and inpatients (Klarström Engström et al., 2019; Verma et al., 2020). Globally, this condition affects approximately 150 million people each year, resulting in approximately 3.5 billion dollars in health costs (Díaz et al., 2020). Furthermore, UTIs can cause serious sequelae, including frequent recurrences, pyelonephritis with sepsis, renal damage in young children, pre-term birth in pregnant women, and complications caused by the frequent use of antimicrobial drugs, including high-level antibiotic resistance (Flores-Mireles et al., 2015; Karavanaki et al., 2019; Rousham et al., 2019; Zhou et al., 2021). Previous research has shown that the focus of infection in 20-30% of all patients with sepsis is localized in the urogenital tract and that urosepsis may cause mortality rates of 25% to 60% in certain patient groups (Wagenlehner et al., 2008).

At present, the gold standard for diagnosing UTIs is still the urine culture, followed by antimicrobial susceptibility testing (AST) using the *clean-catch midstream urine* samples (Sathiananthamoorthy et al., 2019). However, identifying the species of pathogen present, and quantifying the abundance of bacteria present in a given sample by culture is time-consuming and lead to the empirical selection of antibiotics (Arienzo et al., 2019; Zhao et al., 2019; Tien et al., 2020). The clinical guidelines for UTIs indicate that antibiotics should be selected only when the pathogen has been determined (Li et al., 2018). However, this recommendation is rarely implemented in clinical practice (Schmiemann et al., 2010). In order to reduce the empirical antimicrobial regimen of choice and effectively prevent the emergence of multi-drug-resistant uropathogens, it is essential to develop a faster detection method so that we can select targeted drugs in a timely manner.

The aim of this study was to develop a new, rapid, and creative urinary tract infection high-throughput multiplex genetic detection system (UTI-HMGS) to allow the direct detection of 18 uropathogens and simultaneously screening for nine antibiotic resistance genes within only 4 hours. In addition, we determined and validated semiquantitative cutoff values for the UTI-HMGS assay. The clinical application performance of UTI-HMGS was systematically and comprehensively evaluated by detecting clinical urine samples and comparing results with urine culture and Sanger sequencing.

## Methods

### Uropathogens and Resistance Genes

The most common and important 18 causative agents of UTIs and nine relevant antibiotic resistance genes were selected as candidates of the UTI-HMGS assay based on epidemiological investigations and the data from China Antimicrobial Surveillance Network (www.chinets.com), including six Gram-negative bacteria: *Escherichia coli* (*E. coli*), *Klebsiella pneumoniae* (*K. pneumoniae*), *Proteus mirabilis* (*P. mirabilis*), *Pseudomonas aeruginosa* (*P. aeruginosa*), *Acinetobacter baumannii* (*A. baumannii*), *Enterobacter cloacae* (*E. cloacae*); four Gram-positive bacteria: *Enterococcus faecalis (E. faecalis)*, *Enterococcus faecium* (*E. faecium*), *Staphylococcus aureus* (*S. aureus*), *Streptococcus agalactiae* (*S. agalactiae*); and three fungi: *Candida albicans* (*C. albicans*), *Candida glabrata* (*C. glabrata*), *Candida tropicalis* (*C. tropicalis*) (Gajdács and Urbán, 2019; Mario et al., 2019; Medina and Castillo-Pino, 2019). Five difficult to culture pathogens: *Chlamydia trachomatis* (CT), *Ureaplasma urealyticum* (UU), *Mycoplasma hominis* (MH), *Neisseria gonorrhoeae* (NG) and the urinary tract tuberculosis (UTB), which is one of the most common types of extrapulmonary tuberculosis, were also selected for assay (Chien et al., 2017; Xu et al., 2018). Meanwhile, five bacterial (*Staphylococcus epidermidis*, *Staphylococcus hominis*, *Candida parapsilosis*, *Stenotrophomonas maltophilia* and *Serratia marcescens*) that non-pathogenic or infrequent in UTIs were used to validate the accuracy of the UTI-HMGS assay. The resistance genes include four carbapenemase genes (*bla*_KPC_, *bla*_NDM_ *, bla*_IMP_, and *bla*_VIM_), two Extended-spectrum β-lactamases (ESBLs) genes (*bla*_CTX-M_ and *bla*_SHV_), two methicillin resistant-associated genes (*mecA* and *mecC*) of methicillin-resistant Staphylococcus aureus (MRSA) and *vanA*-Type gene of Vancomycin-Resistant Enterococcus (VRE) (Han et al., 2020).

### Urine Specimen Collection

531 urine specimens were collected from outpatients and in patients diagnosed with or suspiciously suffering from UTIs from Huadong Hospital, affiliated with Fudan University. Based on the inclusion criteria, all of the urine samples were collected from the morning midstream clean-catch urine specimens, without considering age and gender of the patients. The urine samples were split into two fractions after received: one was performed quantitative urine culture; one was transferred into a 1.5-mL sterile tube and frozen at -80°C until the nucleic acid extraction. This study has been approved by Huadong Hospital Ethics Committee. The Ethics Approval Number: [2013]-077.

### Uropathogens Culture, Identification and Antimicrobial Susceptibility Testing

The urine culture was performed to obtain the results of identification and colony counts of pathogens according to National Clinical Laboratory Procedures (the fourth version). The urine samples were streaked onto Columbia Blood Agar medium (SHANGHAI COMAGAL MICROBIAL TECHNOLOGY CO., Ltd, Shanghai, China) using a 10-µl inoculation loop. And after at least 24-h incubation at 37°C, the colony counts were performed and recorded. Three or more species are usually reported as ‘mixed culture’ and considered as contaminants (Aspevall et al., 2001). Urine cultures showing a significant growth of a single uropathogen are considered as positive (Chu and Lowder, 2018), and negative urine culture was defined as no growth, insufficient growth or a mixed microbial flora with no predominant organism (Prasada et al., 2019). Significant growth was defined as growth of ≥10^5^ CFU/ml for Gram-negative bacteria and ≥10^4^CFU/mL for Gram-positive bacteria. Significant candiduria was also determined as urine culture growth ≥10^4^ CFU/ml. Bacterial and fungi identification was performed with matrix-assisted laser desorption/ionization time-of-flight mass spectrometry (MALDI-TOF MS) using the VITEK MS. The AST was performed using the VITEK2 COMPACT and the results were interpreted in accordance with the Clinical and Laboratory Standards Institute(CLSI) guidelines (Clinical and Laboratory Standards Institute (CLSI), 2020).

### Primer Design

The 29 pairs of fluorescent dye-labeled primers targeting the specific conserved genomic fragments of the selected 18 uropathogens, nine resistance genes and two control genes (listed above) were designed. Notably, we simultaneously select single-copy gene of each uropathogen for semiquantitative analysis. The human internal DNA control gene (Human DNA) from RP11-320F15 on human chromosome 10, complete sequence and the systematic internal control (IC)–an artificial pseudovirus particles were selected as two quality control genes for UTI-HMGS assay. A large number of sequences for each target were downloaded from National Center of Biotechnology Information (NCBI) and analyzed using Vector NTI (Invitrogen, Carlsbad, USA) to determine the most highly conserved and single copy gene target specific for each uropathogen or resistance gene type. Then the primers that targeted amplification of the highly conserved regions were designed using DNASTAR software (DNASTAR Inc., Madison, WI, USA) and premier 6.0 software (Premier Biosoft International, Palo Alto, CA, USA). All of these primers were designed and optimized according to the following principles: homogeneity of primer sequences; amplification product sizes ranging from 100 to 400 bp, with at least three-base-pair size differences among each target gene fragment; without significant dimer formation between different primers; and absence of non-specific products with each pair of gene-specific primers. The primers sequences, the corresponding amplicon sizes and their targets genes were listed in **Supplementary File 1: Table S1**.

### Plasmid Construction, Transformation, and Purification

The plasmids of all the detection targets for UTI-HMGS assays were used to provide positive control and evaluate the sensitivity for all targets. The construction of 29 plasmids was processed according to the manufacturer’s protocol. DNA copy number of all plasmids was calculated as following formula: [(6.02×10^23^ copy number/mol) × plasmid concentration (g/mL)/L × (MW g/mol) = copies/mL (MW: average molecular weight).

### Sample Pretreatment and Pathogens Nucleic Acid Extraction

The DNA of pathogens and Hum DNA was extracted from 300 µl urine sample using Smart LabAssist –an automatic nucleic acid extraction instrument and its supporting reagents (Taiwan Advanced Nanotech, Taiwan, China) according to the manufacturer’s protocol. Meanwhile, the IC was added to the extraction reagent as internal reference of system quality control. The concentrations of extract were determined using a Thermo Nanodrop 2000 spectrophotometer Thermo Fisher Scientific Inc., Waltham, MA, USA). The DNA samples were stored at -20°C until use.

### Two Multiplex Polymerase Chain Reactions

Both of two multiplex PCR included six components: 2 µl Roche Buffer, 0.5 µl Uracil-DNA Glycosylase (UNG), 0.4 µl FastStart Taq Polymerase, 1 µl primers pool, 1.1 µl ddH_2_O and 5 µl template, the final volume of PCR was 10 µl. The combination of primers for uropathogens panel was consisted of 18 pairs of primers of uropathogens and two control genes, each pair of primer was mixed at different proportion to achieve the optimum sensitivity for all targets. The combination of primers for resistance genes panel included nine pairs of primers for resistance gene and one control gene. Both of two PCR mixture were incubated as follows: 42°C for 5 min; 94°C for 8 min; 94°C for 30 s, 60°C for 30 s, 70°C for 1 min, 34 cycles; 72°C for 1 min.

### Separation by Capillary Electrophoresis and Fragment Analysis

After the multiplex PCR finished, 1 µl of the PCR product was added to 9 µl of highly deionized (Hi-Di) formamide that contained 4% DNA Size Standard 500 (Applied Biosystems, California, USA). Then the PCR products were analyzed by the Applied Biosystems 3500DX genetic analysis system (Applied Biosystems, California, USA) based on size separation that caused by high-resolution capillary gel electrophoresis. The dates obtained above were further analyzed by the GeneMapper ID-X software. Finally, the specific-peak height of PCR product was reported and the results were considered to be positive when the peak height was greater than 500 relative fluorescence units (rfu). The ddH_2_O was simultaneously detected as a negative control throughout the whole experimental process.

### Establishment and Optimization of the UTI-HMGS

Both of uropathogens and resistance genes detection for UTI-HMGS assays were established and optimized as following principle: primer sequences were optimized so that each signature of detection target could be amplified specifically without cross-interaction; other reaction parameters of PCR components and procedures, such as buffer, enzyme, and reaction time, were also systematically optimized. Additionally, considering the presence of the rigid cell wall for gram-positive bacteria and fungi (Gündoğdu et al., 2011; Suriani Ribeiro et al., 2019) against the procedure of nucleic acid extraction, we improved the primers’ concentration of those uropathogens in primer mix. The primers for the Hum DNA and IC were included in the UTI-HMGS PCR primer mix. Detections of Hum DNA in the clinical urine samples indicated that no significant nucleic acid degradation had occurred during specimen handling/storage. The IC (2.5×10^3^ copies in 5 µl) was added to the 300 µL urine samples, immediately prior to nucleic acid extraction, served as internal control for the detection system to monitor the entire process of UTI-HMGS assay. The appearance of both of two internal control peaks in the UTI-HMGS trace confirmed that the urine samples DNA had good integrity and underwent efficient extraction, processing, amplification and capillary electrophoresis.

### Semi-Quantification of the Simulated Positive Urine by UTI-HMGS

The standard strains of 10 species of bacteria and three species of fungi were used to incubate in LB medium at 37°C until visible turbidity. Then bacterial number was quantified by McFarland turbidimetry. After the concentration of initial bacterial suspension was determined, it was tenfold gradient diluted to different concentration of 10^2^ to 10^6^ CFU/mL with urine sample from healthy people. Detection of each concentration was performed by using 300 µl simulated positive urine according to the above method for three replicates. The different gradient concentrations of simulated positive urine were used to validate the quantitative performance of UTI-HMGS.

### Specificity, Sensitivity, and Accuracy of the UTI-HMGS

The specificity of UTI-HMGS assay for ten species of bacteria and three species of fungi were tested with corresponding standard strains. The specificity of remaining five uropathogens, two control genes and nine resistance genes were tested with corresponding plasmids validated by Sanger sequencing. Meanwhile, five negative control pathogens were selected to test the non-specific amplification for uropathogens detection in UTI-HMGS assay. For uropathogens assay, 20 plasmids were mixed by using the equal amounts of templates to attained a mixed plasmid, in which all the plasmids maintain the same concentration. The sensitivity of the UTI-HMGS assay for each pathogen and resistance gene was tested by serial tenfold dilutions of plasmids. Then the serial tenfold dilutions of mixed plasmids using equal amounts of templates were used to test the simultaneous detection limit of UTI-HMGS for all pathogens. And the simultaneous detection limit of UTI-HMGS for all resistance genes was performed following the same methodology above. To further assess the accuracy of the UTI-HMGS, different concentration of three plasmids (*E. cloacae*, 1×10^3^ copies/µL; *K. pneumoniae*, 1×10^2^ copies/µL; *P. mirabilis*, 10 copies/µL) were selected and mixed for testing with the UTI-HMGS assay, and the results were compared with those of single-template UTI-HMSG assay. In addition, the mixed plasmids consist of templates of the five negative control species and three target pathogens (*E. cloacae*, *K. pneumoniae* and *P. mirabilis*) were used to test the performance of the UTI-HMGS assay to accurately identify polymicrobial infection in microbiologically diverse environments.

### Date Analysis and Statistics

The specificity and sensitivity of the diagnostic tests were calculated according to the following formulas: SE=TP/(TP+FN) ×100%; SP=TN/(TN+FP) ×100%; the positive predictive value (PPV) and negative predictive value (NPV) were calculated as follows: PPV=TP/P; NPV=TN/N (FN: false negative; FP: false positive; N: negative; P: positive; SE: Sensitivity; SP: specificity; TN: true negative; TP: true positive). The statistical analysis was analyzed using the Stata statistical software package, version 13.0 (Stata Corp College Station, TX, USA). The difference in the detection sensitivity and rates of different methods were analyzed by the Mann-Whitney rank-sum test and the Kruskal-Wallis H test. A *p*-value of 0.05 was considered significant.

## Results

### The UTI-HMGS Assay Is Highly Specific and Sensitive for the Detection of a Signature Featuring 18 Uropathogens and Nine Resistance Genes

**Figure 1:**
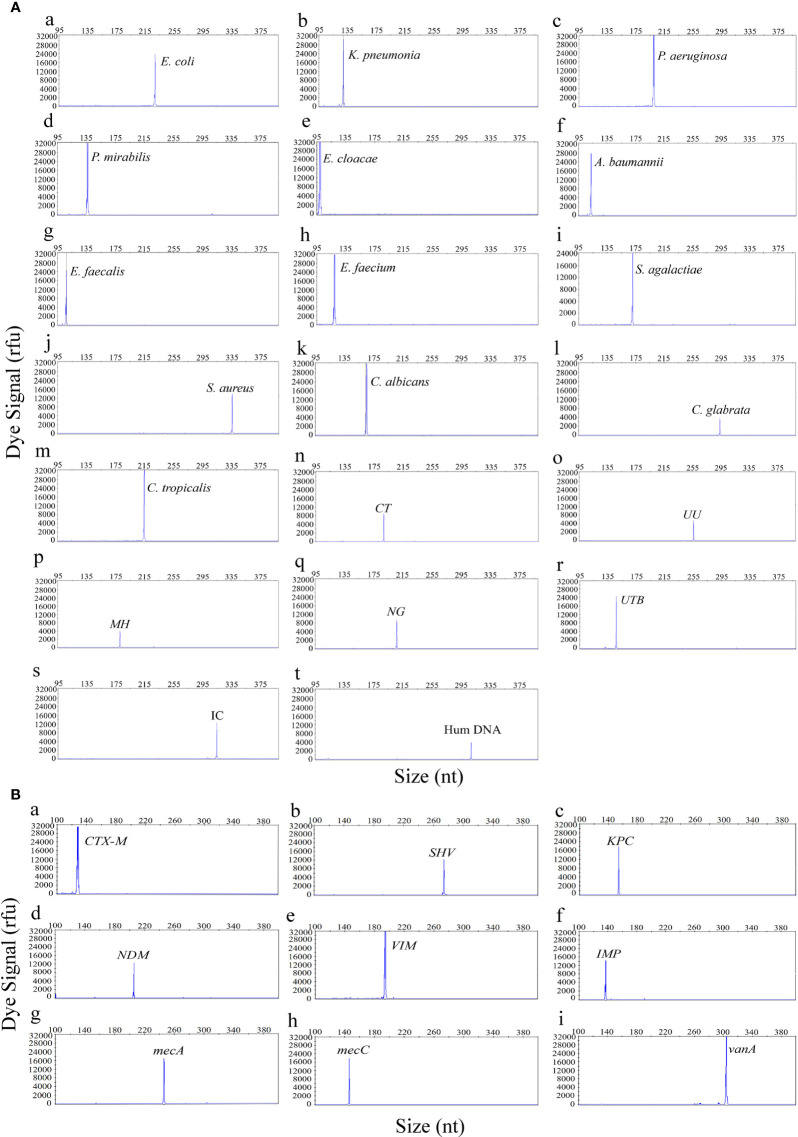
The UTI-HMGS assay produced specific amplification signals for all 29 targets in the panel of uropathogens **(A)** and the panel of resistance genes **(B)**. The X-axis indicates the actual PCR product size while the Y-axis indicates the dye signal. A (a-t) Shows results arising from the amplification of 18 uropathogens and two control genes: *E. coli*, *K. pneumoniae*, *P. aeruginosa, P. mirabilis*, *E. cloacae*, *A. baumannii*, *E. faecalis*, *E. faecium*, *S. agalactiae*, *S. aureus*, *C. albicans*, *C. glabrata*, *C. tropicalis*, CT, UU, MH, NG, UTB, IC and Hum DNA respectively. B (a-i) Shows results arising from the amplification of nine resistance genes: *bla*_CTX-M_, *SHV*, *bla*_KPC_ *, bla*_NDM_, *bla*_VIM_, *bla*_IMP_, *mecA*, *mecC* and *vanA*, respectively. Note that all gene targets were specifically amplified without non-specific amplification by UTI-HMGS.

The first goal of this study was to assess the specificity of the UTI-HMGS for all target genes. The specificity of the UTI-HMGS assay for each uropathogen, resistance gene, and control gene, were validated by using corresponding standard strains or plasmids; these results were then verified by sequencing. This produced specific amplification signals for all 29 targets (**Figure 1**). Under the same experimental conditions, the DNA templates from five pathogens (for which specific primers were not included in our UTI-HMGS assay) were also detected. Results indicated that the UTI-HMGS assay did not produce any specific amplification peaks for these pathogens; similar results were obtained from the negative control ddH2O (**Supplementary File 2: Figure S1**). For all uropathogens, resistance genes, and control genes, the corresponding plasmid of each target was gradient diluted and detected individually by UTI-HMGS. Results showed that the minimum detectable limit for each target was 10 copies/µL; this produced specific peaks that lay above the positive cutoff fluorescence signal of 500 rfu. These results demonstrate the UTI-HMGS exhibits high levels of specificity and sensitivity for the uropathogens, resistance genes, and control genes, tested herein.

### When Optimized, the UTI-HMGS Maintained High Levels of Sensitivity for the Simultaneous Detection of Up to 18 Uropathogens and Nine Resistance Genes in Two Single Multiplex Reactions

**Figure 2:**
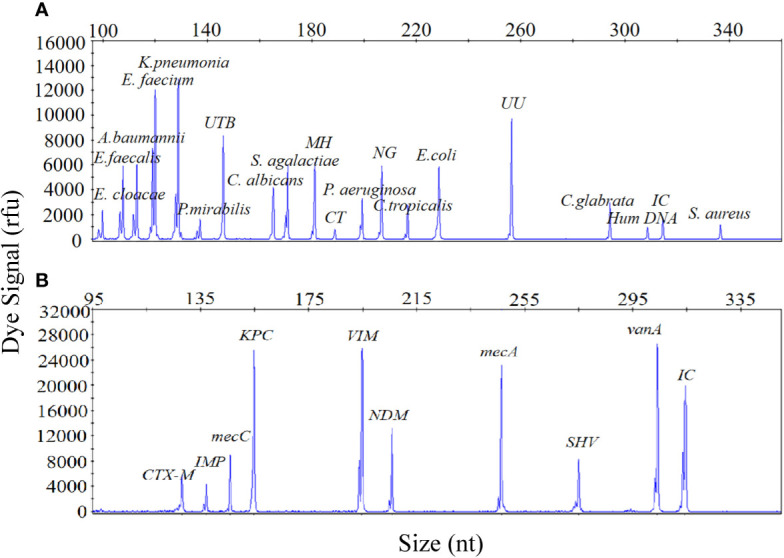
The optimized UTI-HMGS assay maintained high levels of sensitivity for the simultaneous detection of all specific pathogens and control genes tested in the two detection assays. **(A)** The detection limits of the UTI-HMGS assay when detecting uropathogens were determined by amplifying ten-fold diluted plasmids containing equal amounts of 18 uropathogens and 2 quality control templates at a concentration of 100 copies/µL. Note that the uropathogen-defining DNA targets all generated specific peaks (from left to right: *E. cloacae, E. faecalis*, *A. baumannii*, *E. faecium*, *K. pneumoniae*, *P. mirabilis*, UTB, *C. albicans*, *S. agalactiae*, MH, CT, *C. tropicalis*, *E. coli*, UU, *C. glabrata* and *S. aureus*). The quality controls (Hum DNA and IC) produced specific peaks at 308 bp and 315 bp, respectively. **(B)** The detection limit of the UTI-HMGS assay for the resistance genes assay was determined by amplifying tenfold diluted plasmids containing equal amounts of the nine resistance genes and IC at a concentration of 100 copies/µL. Note that the resistance gene-defining DNA targets all generated specific peaks (from left to right: *bla*_CTX-M_, *bla*_IMP_, *mecA*, *bla*_KPC_, *bla*_VIM_, *bla*_NDM_, *mecC*, *bla*_SHV_ and *vanA*). The quality control (IC) produced a specific peak at 315 bp.

The sensitivity of the UTI-HMGS assay was also validated for all targets using a gradient of concentration ranges of a mixture of plasmids created from all of the target plasmids at the same concentration as the uropathogens and resistance genes. As shown in **Figure 2**, the results of the two assays were extremely reliable when detecting 100 copies/µL of the mixture of plasmids; this produced specific peaks above the positive cutoff fluorescence signal of 500 rfu for all targets. Thus, the sensitivity of the UTI-HMGS assay for the simultaneous detection of all uropathogens and resistance genes in two single reactions was at least 100 copies/µL. These results also indicated that the UTI-HMGS assay can simultaneously detect 18 uropathogens or nine resistance genes in a single multiplex reaction.

### The UTI-HMGS Specifically Detected Individual Uropathogens in Polymicrobial Mixtures

**Figure 3:**
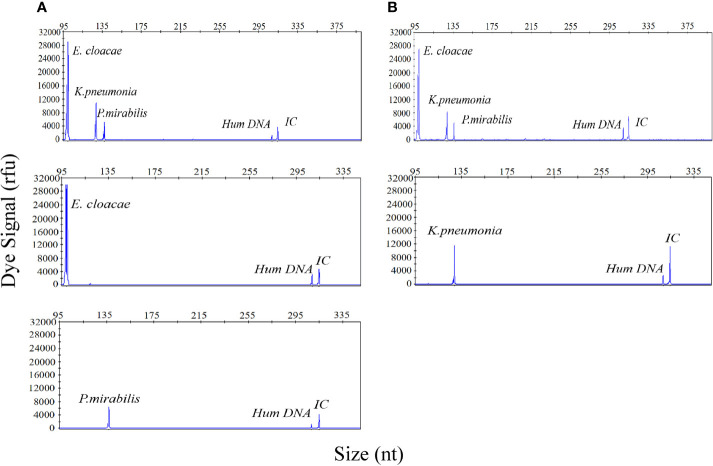
The UTI-HMGS assay accurately detected individual uropathogen in a polymicrobial mixture without interference from other target genes. **(A)** *E. cloacae*, *K. pneumoniae*, and *P. mirabilis* plasmids were combined in different proportions (1×10^3^ copies/µL, 1×10^2^ copies/µL, and 10 copies/µL, respectively); these produced specific peaks at 100 bp, 129 bp, and 138 bp, with high, mid-range and low signal intensities (30,000 rfu, 120,00 rfu, and 6,000 rfu, respectively). **(B)** The combined plasmids of three uropathogens and 5 negative control pathogen templates consistently showed specific peaks for *E. cloacae*, *K. pneumoniae* and *P. mirabilis* with no interference. When tested individually, *E. cloacae* **(C)**, *K. pneumoniae* **(D)** and *P. mirabilis* **(E)** produced specific peaks at 100 bp, 129 bp, and 138 bp, respectively, with corresponding intensities of 30,000, 12,000, and 6,000 rfu. The signals arising from Hum DNA and IC generated peaks at 308 bp and 315 bp, respectively, in each UTI-HMGS reaction.

Considering the incidence of polymicrobial infections in cases of UTI, the ability to detect multiple infections is vital if the UTI-HMGS can be applied effectively in the clinic. To demonstrate this performance, a mixture of three uropathogen-associated plasmids (*E. cloacae* [1×10^3^copies/µL]; *K. pneumoniae* [1×10^2^ copies/µL]; *P. mirabilis* [10 copies/µL]) was detected by the UTI-HMGS assay. As shown in **Figure 3A**, three specific amplification peaks were observed (*E. cloacae*, 100 bp*; K. pneumoniae*, 129 bp; *P. mirabilis*, 138 bp). Furthermore, the addition of six negative control plasmids did not prevent the generation of the specific *E. cloacae*, *K. pneumoniae* and *P. mirabilis* peaks; no false-positive signals were observed in the presence of the negative control plasmids (**Figure 3B**). In the case of polymicrobial infections, it is possible that the various concentrations of templates present may result in significant competition within a single PCR and that the signal produced by a template with low abundance could be weakened by a template that is present in high abundance, thus leading to a false negative result. To test this hypothesis, we detected individual concentrations of each plasmid at the same concentration (*E. cloacae* [1×10^3^copies/µL]; *K. pneumoniae* [1×10^2^ copies/µL]; *P. mirabilis* [10 copies/µL]). Results indicated that the signal intensity produced by the UTI-HMGS assay was completely identical to that produced when using the mixture of plasmids (**Figures 3C–E**). Thus, the UTI-HMGS was able to accurately detect individual uropathogens in polymicrobial mixtures. Moreover, there was no interaction between a dominant DNA template and a low-abundance DNA template.

### UTI-HMGS Semi-Quantitatively Detected 10 Species of Bacteria and Three Species of Fungi in Simulated Positive Urine Specimens

**Figure 4:**
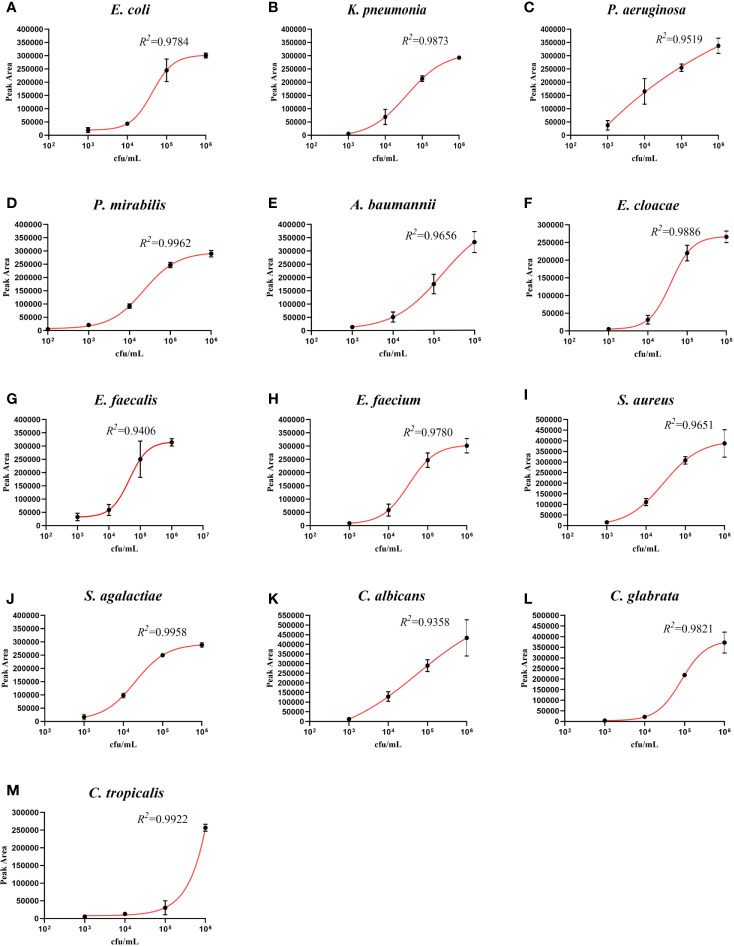
The detection of 13 pathogens in simulated positive urine specimens showing the quantitative standard curves obtained by 10-fold dilutions from 10^2^ to 10^6^ CFU/mL (x-axis) and their corresponding UTI-HMGS peak area (y-axis). Each concentration was tested on three independent occasions using the UTI-HMGS for all 13 pathogens. Error bars are not shown if they are shorter than the size of the symbol used to indicate the mean value of the three peak areas. **(A–M)** Calibration curves for *E. coli* (R^2 =^ 0.9784), *K. pneumoniae* (R^2 =^ 0.9873), *P. aeruginosa* (R^2 =^ 0.9519), *P. mirabilis* (R^2 =^ 0.9962), *A. baumannii* (R^2 =^ 0.9656), *E. cloacae* (R^2 =^ 0.9886), *E. faecalis* (R^2 =^ 0.9406), *E. faecium* (R^2 =^ 0.9780), *S. aureus* (R^2 =^ 9651), *S. agalactiae* (R^2 =^ 0.9958), *C. albicans* (R^2 =^ 0.9358), *C. glabrata* (R^2 =^ 0.9821) and *C. tropicalis* (R^2 =^ 0.9922), respectively.

In order to fully investigate the quantitative performance of the UTI-HMGS assay, 10 species of bacteria and 3 species of fungi were chosen and used to simulate UTIs. Our aim was to identify the concentration of these pathogens that could be used to provide a reference for clinical treatment. The simulated positive urine specimens created from these pathogens at concentrations of 10^2^ to 10^6^ CFU/mL were then detected by the UTI-HMGS assay. Next, we created standard curves for the various concentrations and the corresponding peak areas. As shown in **Figure 4**, the standard curve for each uropathogen in urine showed an increasing tendency upon the increasing concentration of pathogens; the R^2^ values for all 13 pathogens exceeded 0.900. This demonstrated that the UTI-HMGS successfully detected the 13 pathogens in the simulated positive specimen of urine. In addition, the minimum detectable concentration of the 13 pathogens was 10^3^ CFU/mL. Considering that these 13 pathogens all showed significant growth in the positive clinical urine samples collected during our study, it is essential to determine the semi-quantitative UTI-HMGS-based cutoff values for each pathogen so that we can distinguish between positive and negative urine samples from suspected UTI patients. Initially, the semi-quantitative cutoff value for each pathogen was based on the minimum peak area of simulated positive urine samples with 10^5^ CFU/mL for Gram-negative bacteria and 10^4^CFU/mL for Gram-positive bacteria and fungi in the UTI-HMGS assay. When the detection peak area of the UTI-HMGS assay from a 300 µL clinical urine sample exceeded the corresponding semi-quantitative cutoff value for each pathogen, the concentration of pathogen was considered significant (≥10^4^ CFU/mL or 10^5^ CFU/mL) in a urine sample. In other words, the pathogen could be directly and accurately semi-quantified from clinical urine samples by UTI-HMGS. According to the quantitative standard analysis, the semi-quantitative cutoff value were as follows: *E. coli* (206465), *K. pneumoniae* (201686), *P. aeruginosa* (246892), *P. mirabilis* (238441), *A. baumannii* (151176), *E. cloacae* (200032), *E. faecalis* (36860), *E. faecium* (32474), *S. aureus* (93419), *S. agalactiae* (90706), *C. albicans* (106170), *C. glabrata* (12862), and *C. tropicalis* (14093).

### The UTI-HMGS Assay Simultaneously and Semi-Quantitatively Detected Two Pathogens in a Simulated Urine Specimen With a Polymicrobial Infection

**Figure 5:**
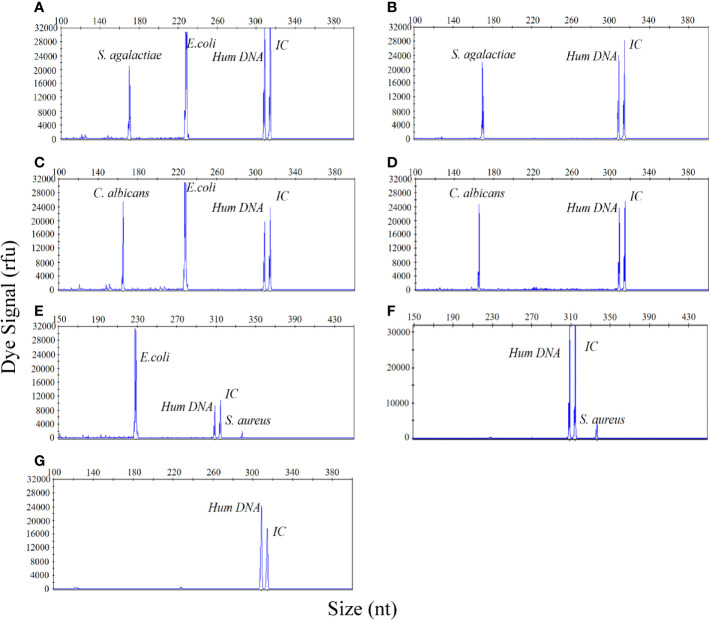
The UTI-HMGS assay can simultaneously and quantitatively detect two pathogens in a simulated dual-infected urine specimen without influence from the other pathogen. **(A)** A simulated dual-infected specimen of urine containing *S. agalactiae* (10^4^ CFU/mL) and *E. coli* (10^5^ CFU/mL). **(B)** A simulated urine sample featuring a single infection of *S. agalactiae* (10^4^ CFU/mL). **(C)** A simulated dual-infected urine specimen containing *C. albicans* (10^4^ CFU/mL) and *E. coli* (10^5^ CFU/mL). **(D)** A simulated urine sample featuring a single infection of *C. albicans* (10^4^ CFU/mL). **(E)** A dual-infected urine specimen containing *S. aureus* (10^4^ CFU/mL) and *E. coli* (10^5^ CFU/mL). **(F)** A simulated urine sample featuring a single infection of *S. aureus* (10^4^ CFU/mL). **(G)** A urine sample acquired from a healthy control without infection (negative control).

In view of the possibility that multiple species of bacteria may be present in urine of patient with a UTI, it is particularly important to be able to quantitatively detect specific species of bacteria without interference from other bacterial species within the same urine specimen. Therefore, specimens of urine with simulated polymicrobial infections, and containing two different pathogens in different quantities, were prepared and detected by our new method. *E. coli* (10^5^ CFU/mL) was chosen as the interfering bacteria to identify whether the detection signal intensities of *S. agalactiae* (10^4^ CFU/mL)*, C. albicans* (10^4^ CFU/mL), and *S. aureus* (10^3^ CFU/mL), were affected by *E. coli.* As shown in **Figures 5A–F**, regardless of the presence or absence of *E. coli* (10^5^ CFU/mL), the signal intensity for *S. agalactiae* (10^4^ CFU/mL)*, C. albicans* (10^4^ CFU/mL) and *S. aureus* (10^3^ CFU/mL), were always consistent irrespective of whether the samples being detected contained a single bacterial infection or a polymicrobial infection. Urine sample without infection was used as a negative control (**Figure 5G**). These results clearly demonstrated that the UTI-HMGS assay can reliably and accurately quantify uropathogens without interference from other pathogens, even when the concentration up to 10^5^ CFU/mL; this is the concentration used as a reference standard for the diagnosis of UTIs.

### Results Arising From the Detection of Uropathogens in Clinical Urine Samples by UTI-HMGS Were in High Agreement With Those Generated by Culture Methods

**Figure 6:**
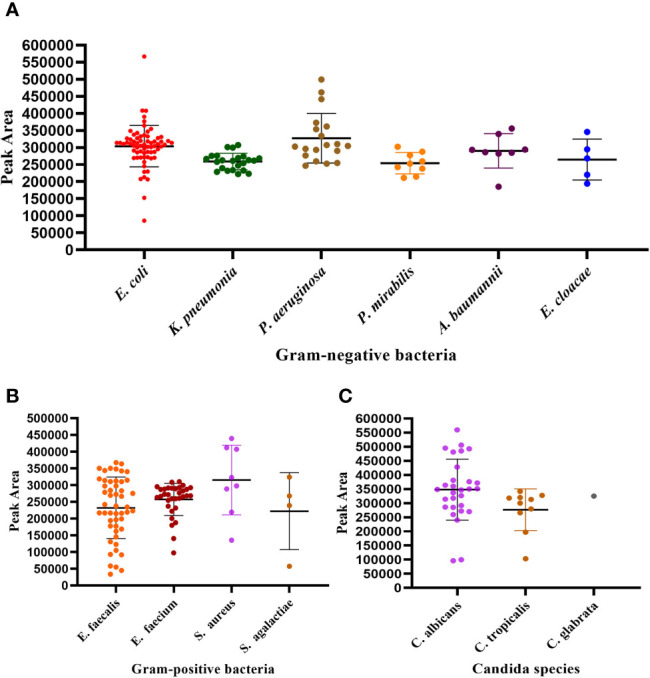
The distribution of peaks for the 13 pathogens by UTI-HGS from the 266 clinical urine samples with monomicrobial infections. **(A)** Peak area distribution of 132 urine samples showing significant growth of one Gram-negative species of bacteria. **(B)** Peak area distribution of 94 urine sample showing significant growth of one species of Gram-positive bacteria. **(C)** Peak area distribution of 40 urine samples showing significant growth of one fungus. The peak area of 261 (98.12%) pathogens from clinical urine samples lay above the corresponding semi-quantitative UTI-HMGS-based cutoff value.

Next, we investigated the performance of the UTI-HMG assay when detecting uropathogens in real clinical samples. For this, we conducted a clinical study by detecting a range of urine samples from 531 patients diagnosed with UTIs or were suspected to be suffering from UTIs. A total of 531 urine samples were collected from these patients and used to validate the detection performance of the UTI-HMGS assay with clinical urine samples. All 531 urine samples involved mid-stream urine (MSU) culture; results showed that 266 cultures were monomicrobial, 25 cultures were polymicrobial and contained two different pathogens, and 240 were negative with no pathogenic infection. Species-level identification by MALDI-TOF MS for the 266 urine samples with monomicrobial cultures were as follows: *E. coli* (n=68), *K. pneumoniae* (n=23), *P. aeruginosa* (n=19), *P. mirabilis* (n=9), *A. baumannii* (n=8), *E. cloacae* (n=5); *E. faecalis* (n=50), *E. faecium* (n=32), *S. aureus* (n=8), *S. agalactiae* (n=4), *C. albicans* (n=29), *C. glabrata* (n=1), and *C. tropicalis* (n=10). Species-level identification tests for the 25 urine samples showing polymicrobial infection, are shown in **Supplementary File 3: Table S2**. Of the 266 monomicrobial cultures, all were correctly identified directly from urine samples by UTI-HMGS (100% concordance rates); 261 were simultaneously semi-quantified (98.12% concordance rates). In other words, the peak area detected by UTI-HMGS for all these pathogens showing significant growth in cultures lay above the corresponding semi-quantitative UTI-HMGS-based cutoff value. The distribution of peaks for the 13 pathogens from the 266 clinical urine samples, as determined by UTI-HMGS, are shown in **Figure 6**. All 25 polymicrobial cultures were simultaneously identified and semi-quantified (100% concordance rates) by UTI-HMGS.

### The Detection of Uropathogens From Clinical Urine Samples by UTI-HMGS Showed High Levels of Specificity and Sensitivity When Compared to the Conventional Culture Method

Next, UTI-HMGS detection results arising from pathogens in the 531 urine samples were compared with the culture-based method. Urine specimens that showed inconsistent results when compared between UTI-HMGS and the conventional culture method were further verified by conventional PCR and Sanger sequencing, widely considered to represent the gold standard for gene detection (Baudhuin et al., 2015; Mu et al., 2016). The primer sequences used to amplify each pathogen by conventional PCR for Sanger sequencing are shown in **Supplementary File 4: Table S3**.

Based on UTI-HMGS results, and subsequent verification by Sanger sequencing, we found that some pathogens showed no apparent growth according to the conventional culture method but produced a specific fluorescence signal when tested by UTI-HMGS (>500 rfu); in these cases, the peak area lay above or far below the semi-quantitative cutoff values. Of the 266 clinical urine samples with a monomicrobial culture, 181 samples (68.25%) were only detected the culture-positive pathogen and the two control genes; 17 samples (6.39%) were detected 1-3 other pathogens by UTI-HMGS with the detection peak areas lying above the cutoff values but classified as culture negative; 62 samples (23.31%) were detected one to four other pathogens by UTI-HMGS with the detection peak areas lay far below the cutoff values and were classified as culture negative. In addition, in 11 (4.14%) urine samples, UTI-HMGS detected three different difficult-to-culture pathogens, including NG (4), MH (4) and UU (3) (**Supplementary File 5: Table S4**). Of the 25 clinical urine samples, apart from 14 (56%) were only detected the culture-positive pathogens and two control genes by UTI-HMGS, UTI-HMGS detected culture-negative pathogens with a peak area below the assigned cutoff values from 2 urine samples and detected another 1-2 pathogens with peak areas that lay above the cutoff values from 6 urine samples (**Supplementary File 3: Table S2**). Of the 240 clinical urine samples that were negative according to conventional culture methods, 116 (48.33%) samples only showed peaks for Hum DNA and IC in the UTI-HMGS; 13 (5.42%) were identified to be infected by 1-2 pathogens with peak areas above the corresponding semi-quantitative cutoff values; 92 (38.33%) were identified to be infected by 1-3 pathogens with peak areas far below the corresponding semi-quantitative cutoff values. In 30 urine samples, UTI-HMGS detected 1-2 different difficult-to-culture pathogens, including UU (18), CT (4), UTB (3), NG (2), MH (1) and UU+MH (2) (**Supplementary File 6: Table S5**). Finally, the nucleic acids extracted from all clinical urine samples were used for DNA sequencing; this is the most stringent method to calculate sensitivities, specificities, and the predictive values of UTI-HMGS to compare with the culture method. The rate of complete agreement between the culture method and UTI-HMGS was 67.01% (195/291) for 291 urine culture-positive samples (266 monomicrobial cultures and 25 polymicrobial cultures), 48.33% (116/240) for 240 culture-negative samples, and 58.57% (311/531) for all 531 urine samples collected in our study. These results consistently showed that the sensitivity and NPV were higher for the UTI-HMGS assay than for the culture-based method for all uropathogens (**Tables 1** and **2**).

**Table 1:** A comparison of conventional methods and Sanger sequencing for the detection of individual *uropathogens*.

Pathogens	Culture	Sequencing		Sensitivity	Specificity	PPV	NPV	Accuracy
		+	−					
*E. coli*	**+**	79	0	0.622	1.000	1.000	0.894	0.910
	**−**	48	404			
*K. pneumoniae*	**+**	26	0	0.456	1.000	1.000	0.939	0.942
	**−**	31	474					
*P. aeruginosa*	**+**	23	0	0.561	1.000	1.000	0.965	0.966
	**−**	18	490					
*P. mirabilis*	**+**	10	0	0.555	1.000	1.000	0.985	0.985
	**−**	8	513					
*A. baumannii*	**+**	8	0	0.571	1.000	1.000	0.986	0.989
	**−**	6	517					
*E. cloacae*	**+**	5	0	0.417	1.000	1.000	0.987	0.987
	−	7	519					
*E. faecalis*	**+**	64	0	0.621	1.000	1.000	0.916	0.927
	**−**	39	428					
*E. faecium*	**+**	40	0	0.563	1.000	1.000	0.937	0.942
	**−**	31	460					
*S. aureus*	**+**	8	0	0.727	1.000	1.000	0.994	0.994
	**−**	3	520					
*S. agalactiae*	**+**	5	0	0.455	1.000	1.000	0.989	0.989
	**−**	6	520					
*C. albicans*	**+**	34	0	0.523	1.000	1.000	0.938	0.942
	**−**	31	466					
*C. tropicalis*	**+**	10	0	0.769	1.000	1.000	0.994	0.994
	**−**	3	518					
*C. glabrata*	**+**	4	0	0.364	1.000	1.000	0.987	0.987
	**−**	7	520					

The sensitivity of the culture-based method was lower and more variable for a range of uropathogens.

**Table 2:** UTI-HMGS exhibited uniformly high sensitivity for the detection of uropathogens when compared to the Sanger sequencing method.

Pathogens	HMGS	Sequencing		Sensitivity	Specificity	PPV	NPV	Accuracy
		+	−					
*E. coli*	**+**	125	2	1.000	0.995	0.984	1.000	0.996
	**−**	0	404			
*K. pneumoniae*	**+**	56	1	1.000	0.998	0.982	1.000	0.998
	**−**	0	474					
*P. aeruginosa*	**+**	41	0	1.000	1.000	1.000	1.000	1.000
	**−**	0	490					
*P. mirabilis*	**+**	18	0	1.000	1.000	1.000	1.000	1.000
	**−**	0	513					
*A. baumannii*	**+**	14	0	1.000	1.000	1.000	1.000	1.000
	**−**	0	517					
*E. cloacae*	**+**	12	0	1.000	1.000	1.000	1.000	1.000
	−	0	519					
*E. faecalis*	**+**	103	2	1.000	0.995	0.981	1.000	0.996
	**−**	0	426					
*E. faecium*	**+**	71	1	1.000	0.998	0.986	1.000	0.998
	**−**	0	459					
*S. aureus*	**+**	11	0	1.000	1.000	1.000	1.000	1.000
	**−**	0	520					
*S. agalactiae*	**+**	11	0	1.000	1.000	1.000	1.000	1.000
	**−**	0	520					
*C. albicans*	**+**	65	1	1.000	0.998	0.985	1.000	0.998
	**−**	0	465					
*C. glabrata*	**+**	13	0	1.000	1.000	1.000	1.000	1.000
	**−**	0	518					
*C. tropicalis*	**+**	11	0	1.000	1.000	1.000	1.000	1.000
	**−**	0	520					
CT	**+**	4	0	1.000	1.000	1.000	1.000	1.000
	**−**	0	527					
UU	**+**	23	0	1.000	1.000	1.000	1.000	1.000
	**−**	0	508					
MH	**+**	7	0	1.000	1.000	1.000	1.000	1.000
	**−**	0	524					
NG	**+**	6	0	1.000	1.000	1.000	1.000	1.000
	**−**	0	525					
UTB	**+**	3	0	1.000	1.000	1.000	1.000	1.000
	**−**	0	528					

The UTI-HMGS assay showed high levels of sensitivity and was comparable to the Sanger sequencing method for all of the uropathogens tested.

### The Clinical Application of UTI-HMGS Improved the Positive Detection Rates for All Uropathogens and Showed That Routine Clinical Cultures Missed the Detection of Some Important Uropathogens

**Figure 7:**
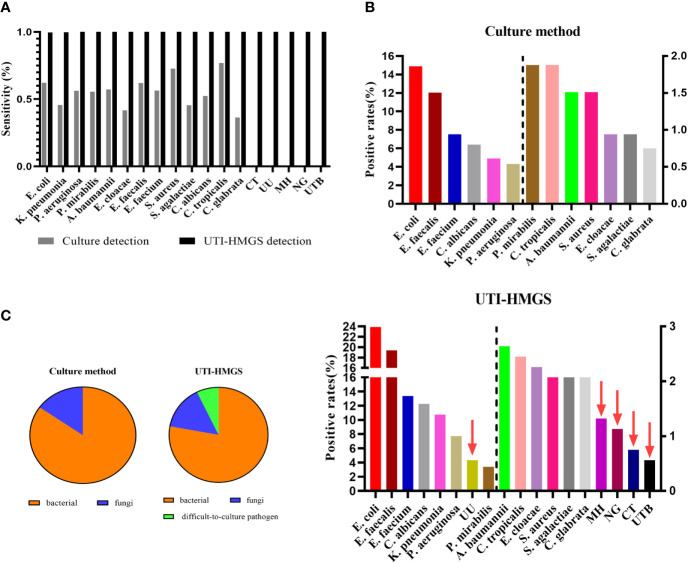
The uniformly high sensitivity of the UTI-HMGS assay improved positive detection rates and revealed the missed detection of some important uropathogens by routine urine cultures in the clinic. **(A)** A comparison of the culture method *versus* the UTI-HMGS assay with regard to the sequencing-based detection of 18 uropathogens. The sensitivity of the UTI-HMGS assay for the detection of *E. coli, K. pneumoniae* (*P*=0.00), *P. aeruginosa* (*P*=0.00), *P. mirabilias* (*P*=0.00), *A. baumannii* (*P*=0.01), *E. cloacae*(*P*=0.01), *E. faecium* (*P*=0.00), *E. faecalis* (*P*=0.00), *S. agalactiae*(*P*=0.01), *C. albicans* (*P*=0.00), *C. glabrata* (*P*=0.01), CT (*P*=0.04), UU (*P*=0.00), MH (*P*=0.01) and NG (*P*=0.01) were significantly higher than that of the culture method **(B)** The positive detection rates of individual uropathogens ranked according to detection results from the culture method (top) and the UTI-HMGS assay (bottom). The UTI-HMGS assay improved the positive detection rates attained by the culture method and revealed the missed detection of UU, MH, CT and UTB by culture. **(C)** The uniform sensitivity of the UTI-HMGS assay when detecting bacteria, fungi, and difficult-to-culture pathogens, altering the ratios of 13 pathogens that can be cultured *versus* five difficult-to-culture pathogens, thus underlining the importance of comprehensive detection in the diagnosis of UTIs.

In our earlier analyses, we identified a notable difference between the conventional culture method and the UTI-HMGS assay for the detection of clinical samples; some samples were negative for pathogens when tested by conventional culture but were shown to produce a specific fluorescence signal when tested by UTI-HMGS (>500 rfu). Consequently, we analyzed the positive detection rate for each pathogen from both the culture method and the UT-HMGS assay. A comparison of the detection rates by the conventional culture method and the UTI-HMGS assay for 18 uropathogens demonstrated that the sensitivity of the UTI-HMGS for 15 of them were significantly higher (*P*<0.05) than that of the culture-based method (**Figure 7A**). Our analysis showed that CT, UU, NG, MH, and UTB were perceived as difficult-to-culture pathogens in our clinical laboratory tests and were not detectable by the routine culture method. The UTI-HMG assay did not only detect 13 cultivable uropathogens, the new method also simultaneously detected the five difficult to culture pathogens, thus providing a more comprehensive diagnostic screen. Based on the positive cutoff fluorescence signal of 500 rfu for the UTI-HMGS assay, it was evident that the positive detection rates of individual uropathogens were all improved for all uropathogens when compared with the conventional culture method (**Figure 7B**). In particular, of the 531 clinical urine samples that yielded positive or negative cultures, a total of 41 (41/531, 7.72%) were shown to be infected by difficult-to-culture pathogens by UTI-HMGS; these were not detected by the conventional culture method (**Figure 7B**). The difficult-to-culture pathogens were completely missed by culture in our clinical laboratory tests but were successfully detected by UTI-HMGS; this means that the composition of pathogens from 531 urine samples were altered by UTI-HMGS (**Figure 7C**). These results indicated that the high sensitivity of detection achieved by the UTI-HMGS assay can make up for the defects associated with the conventional culture method and thus provide a more comprehensive diagnostic test for uropathogens. This highlights the importance of the UTI-HMGS assay as powerful tool for clinical detection.

### The Detection of Resistance Genes From Clinical Urine Samples by UTI-HMGS Exhibited Extremely High Accuracy Compared to the Sequencing Method

The UTI-HMGS assays could simultaneously detect uropathogens and resistance genes in the same clinical urine samples. According to the results of AST, we detected 36 drug resistance phenotype positive samples, including 24 *ESBLs*-producing *E. coli*, 4 Carbapenemase-resistant *P. aeruginosa*, 5 Carbapenemase-resistant *K. pneumoniae* (CRKP) and 3 *ESBLs*-producing *P. mirabilis.* While detecting 18 uropathogens, UTI-HMGS was also used to detect nine resistance genes in clinical urine samples. Results showed that one drug resistance gene was simultaneously detected in 21 samples, two drug resistance genes were simultaneously detected in 8 samples, three drug resistance genes were simultaneously detected in 1 sample, and no drug resistance genes were detected in 6 samples (these 6 samples only showed a peak for IC) (**Table 3**). In addition, in view of the fact that MRSA and VRE were not found in any of the clinical samples by AST, we used 6 VRE strains and 27 MRSA strains that were isolated from clinical samples of patients with other infectious diseases to prepare simulated infected urine samples (10^4^ CFU/mL); of these, the UTI-HMGS assay detected 4 *vanA* genotypes and 26 *mecA* genotypes. Based on the results of Sanger sequencing, the sensitivity and specificity of the UTI-HMGS assay were both 100% for a total of 69 urine samples (**Table 4**). Data showed that 86.96% (60/69) of urine samples with a positive drug resistance phenotype also harbored corresponding drug resistance gene types. These results demonstrated that the UTI-HMGS exhibited excellent detection performance for drug-resistance genes that are clinically important.

**Table 3:** UTI-HMGS results for 36 positive urine samples that showed a drug-resistant phenotype.

Species (n)	Detection results arising from the UTI-HMGS assay (n)
*ESBLs*-producing *E. coli* (24)	*bla*_CTX-M_ (17)
	*bla*_CTX-M_ and *bla*_SHV_ (2)
	*bla*_SHV_ and *bla*_KPC_ (1)
	*bla*_CTX-M_, *bla*_KPC_ and *bla*_SHV_ (1)
	negative (3)
Carbapenemase-resistant *P. aeruginosa* (4)	*bla*_KPC_ (2)
	*bla*_KPC_ and *bla*_SHV_ (2)
Carbapenemase-resistant *K. pneumoniae* (5)	*bla*_KPC_ and *bla*_SHV_ (3)
	negative (2)
*ESBLs*-producing *P. mirabilis* (3)	*bla*_CTX-M_ (2)
	negative (1)

**Table 4:** UTI-HMGS exhibited uniformly high rates of accuracy for the detection of antimicrobial resistance genes when compared to the Sanger sequencing.

Resistance genes	HMGS	Sequencing		Sensitivity	Specificity	PPV	NPV	Accuracy
		+	−					
*bla*_CTX-M_	**+**	22	0	1.000	1.000	1.000	1.000	1.000
	**−**	0	47			
*bla*_SHV_	**+**	9	0	1.000	1.000	1.000	1.000	1.000
	**−**	0	60					
*bla*_KPC_	**+**	9	0	1.000	1.000	1.000	1.000	1.000
	**−**	0	60					
*mecA*	**+**	26	0	1.000	1.000	1.000	1.000	1.000
	**−**	0	43					
*vanA*	**+**	4	0	1.000	1.000	1.000	1.000	1.000
	**−**	0	65					

## Discussion

UTIs are one of the most common infections that affects human beings worldwide. The culture of urine samples remains the gold standard for diagnosing UTIs, but test sensitivity is poor and turn-around time is slow. This increases the non-standard use of antibiotics and accelerates the emergence of drug-resistant bacteria. In addition, the culture method may not be able to cover all species of pathogens, especially for difficult-to-culture pathogens, causing the missed detection of some important pathogens. This delays the diagnosis and treatment of patients. In order to improve the current status of diagnosis and treatment for UTIs, it is necessary to develop new technology.

In this research, we present a new molecular approach (UTI-HMGS) that allows the direct detection of 18 pathogens and simultaneous screening for nine resistance genes in urine specimens to facilitate the diagnosis of UTIs. We investigated the diagnostic performance of the UTI-HMGS with different experimental designs to highlight the potential benefits of this new method in comparison with conventional urine culture, considered as the gold standard. The results reported herein demonstrate the overall high accuracy, specificity, and sensitivity, of the UTI-HMGS assay.

In our study, we preferentially selected the most common and important uropathogens based on the prevalence of pathogens that are associated with UTIs in China. According to the data from China Antimicrobial Surveillance Network (http://www.chinets.com/), the most common 10 bacteria species leading to UTI were all covered in our method, and 90.07% bacteriuria were caused by these 10 bacteria in China in 2020. For carbapenemase gene, the most prevalent carbapenemase gene were *bla*_KPC_ and *bla*_NDM_, accounted for 51.6% and 35.7%, respectively in all carbapenem-resistant Enterobacteriaceae (CRE) strains isolated from 2016 to 2018 across China (Han et al., 2020). Since 2000, CTX-M β-lactamases have been identified as the most widespread ESBL (Jung et al., 2020). The mecA and mecC were both included in UTI-HMGS for the surveillance of MRSA, which is a leading cause of deadly hospital-acquired infections (Lamontagne Boulet et al., 2018). For the resistance genes related to VRE, the most widely distributed in clinical strains is vanA in a lot of studies, especially in China (Dehbashi et al., 2020). Apart from the most common and important pathogens or resistance genes above, the number of remaining uncommon species and resistance genes were too large that it is not feasible to cover all of them in UTI-HMGS. But for better clinical diagnosis, we will include the other uncommon pathogens and resistance genes in our method if one day they become prevalent.

In particular, our new method is able to identify five important pathogens that are difficult to culture under routine laboratory conditions but could cause significant harm to patients, including CT, UU, MH, NG, and UTB. The frequent lack of obvious symptoms in patients with urinary tract infections caused by these pathogens is one major reason for introducing new screening programs for their improved detection. NG is the etiologic agent of gonorrhea and gonococcal urethritis, one of the most frequent cause of bacterial sexually transmitted infections (STIs) worldwide (Jiang et al., 2017).

The most significant consequences of CT, UU and MH infections in females include the risk of infertility, ectopic pregnancy, and pelvic inflammatory diseases (Liu et al., 2019). According to previous reports, it is estimated that symptomatic carriers of NG and CT have a relative risk of 4.8-fold and 3.6-fold, respectively, for the sexual acquisition of HIV (Silva et al., 2018). Until recently, the diagnosis of CT infections depended on cell culture techniques as the gold standard for the detection of pathogens in clinical specimens (El-Sayed et al., 2006). However, this pathogen does not normally grow outside living cells; this poses a significant challenge with regards to diagnosis. Genitourinary tuberculosis (GUTB) is the second most common form of extrapulmonary tuberculosis; previous research showed that more than 90% of cases occurred in developing countries (Abbara and Davidson, 2011). The gold standard for the diagnosis of GUTB involves the isolation and culture of Mycobacterium tuberculosis (Nataprawira et al., 2019). However, the diagnosis of GUTB is often delayed because the clinical features of this disease are non-specific and there is a long latency period between initial TB infection and the onset of symptoms (Abbara and Davidson, 2011). In the present study, we developed a method that can directly detect five difficult-to-culture uropathogens above from urine specimens with a minimum detection limit of 50 copies per reaction. Our validation tests showed that our new method was highly accurate when compared with Sanger sequencing (**Table 2**), thus indicating that the UTI-HMGS assay represents a potential method to monitor these important uropathogens.

The most important difference between urine samples and other clinical samples (such as blood) in the diagnosis of pathogens is the need for specific quantification when diagnosing a case of UTI. Previous studies reported that bacterial loads below 10^5^ CFU/mL were considered to be UTI-negative while bacterial loads above 10^5^ CFU/mL were considered to be UTI-positive or diagnosed as having significant bacteriuria (Hansen et al., 2013; Belete et al., 2019; Gebremariam et al., 2019; Karikari et al., 2020). Other studies opted to use lower colony counts of >10^4^ CFU/mL as an indicator of significant bacteriuria (Milovanovic et al., 2019; Schuh et al., 2019). In our study, we considered the fact that the growth rate of Gram-positive bacteria is slower than that of Gram-negative (Alpaslan et al., 2017); thus, a load of 10^5^ CFU/mL was defined as significant bacteriuria for Gram-negative bacteria while a load of 10^4^ CFU/mL was defined as significant bacteriuria for Gram-positive bacteria. In order to detect uropathogens in a semi-quantitative manner, we selected a single-copy gene for each uropathogen and limited the urine test volume to 300 µL while we established and validated our UTI-HMGS assay with actual clinical urine samples. In a fixed volume of urine, we observed excellent levels of correlation between the detection peak area of the UTI-HMGS assay and the gradient concentration for all 13 uropathogens (**Figure 4**). Based on these findings, we determined a semi-quantitative UTI-HMGS-based cutoff value for each pathogen to identify whether the concentration of a given pathogen was significant (≥ 10^4^ CFU/mL or 10^5^ CFU/mL) in a 300 µL urine sample (simulated). Finally, the semi-quantitative cutoff values were further validated by the use of actual clinical urine samples; these yielded 98.81% concordance rates when compared with the colony count derived from midstream urine cultures (**Figure 6**). These results show that the UTI-HMGS was reliable and stable for practical application in the clinic. In addition, five difficult-to-culture uropathogens were detected successfully with our semi-quantitative assay, as based on the concordance between the copy number of pathogens and the corresponding peak area detected by the UTI-HMGS assay (*date not shown*). However, considering we only had a limited number of positive urine samples in the present study, we were not able to fully validate the correlation between copy number and peak area in this study.

An alternative method that could be used to further improve the sensitivity of UTI-HMGS for the 18 uropathogens and nine resistance genes tested herein is to centrifuge the urine samples prior to analysis as this can cause an enrichment of pathogens from larger volumes of urine (1-10 mL). However, in the present study, the minimum detection limit of the UTI-HMGS assay for the most common uropathogens was 10^3^ CFU/mL. This detection limit fully meets the clinical needs for UTI diagnosis. Our method is also simple to perform in a clinical scenario. Thus, in the present study, we did not incorporate a concentration step, even though it can be argued that this step may have been beneficial as it could have increased the overall sensitivity of the assay.

As the UTI-HMGS assay is based on PCR, a notable difference between our method and the culture-based method could be the detection of DNA from dead uropathogens; this scenario could promote the detection of false positive results. We identified urine samples that were culture-negative but showed peak areas that were above the corresponding semi-quantitative cutoff values derived from dead uropathogens. However, the proportion of such urine samples in our study was very low (13 out of 240 [5.42%] clinical urine samples showed a negative culture result while 17 out of 266 [6.39%] clinical urine samples showed monomicrobial infection). Consequently, the detection of DNA from dead uropathogens appears to be very limited. On the other hand, in order to diagnose UTIs and determine whether drug intervention is necessary, it is important to evaluate urinary symptoms *via* urinalysis in order to screen for the presence of white blood cells, leukocyte esterase, and nitrites (Berends et al., 2019; Broeren et al., 2019). Such information is vital if we are to judge accurately whether the pathogen detected by UTI-HMGS represents an active infection that requires intervention.

The plasmid-mediated transfer of resistance genes among bacterial species is recognized as one of the most important mechanisms that can accelerate the dissemination and emergence of drug resistance (Bai et al., 2017; Bharathan et al., 2019) *via* the β-lactamase gene, the carbapenem resistance gene, VRE, and MRSA drug resistance (Lin and Hayden, 2010; Sultan et al., 2018; Salah et al., 2019; van Beek et al., 2019). These factors increase the frequency of resistant strains and increase the threat for modern medicine. Preventing the spread of resistance genes relies on isolation precautions and antibiotic stewardship (Lin and Hayden, 2010; Chalfine et al., 2012). The simultaneous detection and discovery of resistance genes by UTI-HMGS is critical for preventing the spread of plasmids related drug resistance, can prevent an increase in bacterial resistance and reduce the use of non-standard antibiotics. For these reasons, we believe the future clinical application of the UTI-HMGS assay could make important contributions to the prevention, control, and treatment, of clinical nosocomial infections.

The limitations of this study are the following. Firstly, the inclusion of clinical positive samples all had significant bacteriuria or candiduria (10^4^CFU/mL or 10^5^CFU/mL). Thus, the semi-quantitative cutoff value obtained by the simulated urine with other concentration levels were not validated by clinical urine samples due to the fact that these are extremely rare in our clinical laboratory tests. Secondly, the detection cost of UTI-HMGS is about $5 per urine sample, in other words, about $0.2 per target, which will increase the detection costs of the diagnosis of UTIs compared with conventional culture. However, considering our method is able to provide a comprehensive insight of potential pathogens in urine samples and drug resistance information within only 4 hours, we believe that UTI-HMGS could be of great value to improve the diagnosis and therapy of UTIs, especially for blocking the rise of drug-resistant strains.

## Conclusion

We developed and optimized a rapid, specific, and highly sensitive multiplex assay (UTI-HMGS) for the identification of uropathogens and resistance genes. Our assay was able to identify 18 pathogens and nine resistance genes directly from clinical urine samples within 4 hours. The systematic analysis of 531 clinical urine specimens using UTI-HMGS-based semiquantitative cutoff values determined by simulated urine samples, was able to accurately identify positive urine samples with significant bacteriuria or candiduria. In summary, the UTI-HMGS assay could be of significant value for the diagnosis and therapy of UTIs. Our assay could prevent the use of non-standard antibiotics and favor the standard use of prophylactic antibiotics thus avoiding the emergence and spread of drug-resistant bacteria.

## Data Availability Statement

The raw data supporting the conclusions of this article will be made available by the authors, without undue reservation.

## Author Contributions

ZS, WL, and JZ contributed equally to this work equally. WL, JZ, SW, FY, YF, WJ, LD, HZ, and YZ contributed to the design and coordinated the study. ZS, WL, and JZ collected the samples. ZS performed the experiments, analyzed the data. ZS, HZ, and YZ wrote the manuscript. All authors contributed to the article and approved the submitted version.

## Funding

This work was supported by the Shanghai Science and Technology Committee (grant 18411960600); Fudan University excellence 2025—Outstanding talents (grant H1435); Shanghai Science and Technology Committee (grant 18411950800); National Natural Science Foundation Grant of China (grant 81602072);Shanghai Science and Technology Committee “Lead project” (grant 16411968000); Shanghai Shenkang Hospital Development Center “New frontier technology joint research project”(grant SHDC12015107); Ministry of Science and Technology “The National High Technology Research and Development Program of China (863 Program)”(grant 2015AA021107-019); Shanghai “medical star” young medical talents training funding plan (outstanding young medical talents) (HWJ HR [2019] No.72);”Huadong Hospital project”(grant 2019jc020, 2019jc019).

## Conflict of Interest

The authors declare that the research was conducted in the absence of any commercial or financial relationships that could be construed as a potential conflict of interest.
