# Supplementary material for: The Direct Semi-Quantitative Detection of 18 Pathogens and Simultaneous Screening for Nine Resistance Genes in Clinical Urine Samples by a High-Throughput Multiplex Genetic Detection System
Source: Front Cell Infect Microbiol. 2021 Apr 12;11:660461. doi: 10.3389/fcimb.2021.660461 (PMC8072482; doi:10.3389/fcimb.2021.660461)
Supplement: Supplementary file 1 [file DataSheet_1.doc]

Supplementary Material

**Supplementary file 1: Table S1** Primer sequences and product size in UTI-HMGS

| Targets | sequences (5′→3′) | size(bp) |
| --- | --- | --- |
| *Uropathogens* |  |  |
| *E. coli* | F: TCTGTCAACGCTCTACAGTTCCGGTAAGACGC | 228 |
|  | R: GTATGCCCATATTGAAGCGCCGTCCAGTAA |  |
| *K. pneumonia* | F: TCTGTCCAATAGATTTCAGAATGCGGAGTCGTTCA  R: GTATGCCCATATACGCGTCGATGCCACTCGGCC | 129 |
| *P. mirabilis* | F: TCTGTCTGAATATTCCTCACGCCCTGAATATTGTC  R:GTATGCCCATATATCTGATCGGTCAATTCCCAATGTA | 138 |
| *P. aeruginosa* | F: TCTGTCAACTAGTGTTCAACCGGACCTGTGG  R: GTATGCCCATAATGGTTGCCTGGTAGTCTTCGG | 199 |
| *A. baumannii* | F: TCTGTCAACACAACGATTAGCAACTCGTCTTAGCT  R:GTATGCCCATATTTAAACGTTGCTGAGCATACGGAA | 113 |
| *E. cloacae*  *E .faecalis* | F: TCTGTCAACTGCATTCGCGAAATGTCAGATAACGA  R: GTATGCCCATCTTACTCATGTTCGTTACCCATCCAC  F: TCTGTCAACCTATTCCGATGTTAGGTGTCGTAACT | 100  107 |
|  | R: GTATGCCCATAAATCAGGAGGTAACGGGAAGAAAT |  |
| *E. faecium* | F: TCTGTCAACAATGGGCTATGATGTAACGATGACG  R: GTATGCCCATTAGGTAGCCAAGCTGAAACCATGGC | 120 |
| *S. aureus* | F: TGAAGGGTGCATTATGGACGTTAGCGC  R: GTCGTGCTTGCGGATACATATTCTCTGC | 336 |
| *S. agalactiae* | F: TCTGTCAACTACGACGTCACGCTACATCCAA  R: GTATGCCCATGTAACGTCCCTTGTTTCCCTTGC | 172 |
| *C. albicans* | F: TGTCAAGTCCAACAGAAACCCAAATCG | 164 |
|  | R: GTAGGCAAATAAGCGTAGATCCCATAG |  |
| *C. glabrata* | F: TGATCTCCGAGATGTCATAGCCCTTTA  R: GTAGACTCGGCTCCTGTTTAACTGAAT | 293 |
| *C. tropicalis* | F: TGACTATATCGTCGTCGCGTCTGAATT | 216 |
|  | R: GTAGGTAGACGCGGAACTCAATAGAACT |  |
| *CT* | F: AGGGCTTCCTTACCCATAAACTTACGCA  R: ATGCTGTGACTTGTTGTAGTGTGTGAA | 189 |
| *UU* | F: TGCCATAGAAATGCACGAGATTACAA | 257 |
|  | R: GTTGGTGTACCATTCCAATACCAGTT |  |
| *MH* | F: CGCCCATAAGTGCCTTACCAAGTATA  R: TCTGATACCGCAACCGCTATTGTATAC | 181 |
| *UTB* | F: TGAATGCGAGCTCGAGGGATACCAGT  R: GTCCAGTAGAGGACGCCAAGAGGGT | 147 |
| *NG* | F: TATCGATGCGGACACCCAATACCTGC  R: GTTTGAAATCTCCGTTGCCCATACCGG | 206 |
| *Hum DNA*  *IC* | F: TGTCCGTGCCCCAAATTCCAG  R: GTCTCGGTCAGGTCTCCCA  F: TTGATGGCACAGTCGAGGCTG  R: GTGGCCGCTTTTCTGGATTCAT | 308  315 |
| resistance genes |  |  |
| *CTX-M* | F: TGCAGCACCAGTAAAGTKATGGC  R: GTTTCGGCAATCGGATTRTAGTT | 128 |
| *SHV*  *KPC* | F: TTGCGTTATATTCGCCTGTGTATTATCTC  R: GTGCTGGCGATAGTGGATCTTTC  F: ACTGTGCAGCTCATTCAAGG  R: GTCGTCATGCCTGTTGTCAGA | 275  154 |
| *NDM* | F: TCCATTAGCCGCTGCATTGA  R: GTCCATCCCTGACGATCAAAC | 205 |
| *IMP* | F: ATTGACACTCCATTTACDGCTAAAGA  R: GTCGAGAATTAAGCCACTCTATTCC | 137 |
| *VIM* | F: TGTCCGTGATGGTGATGAGTT  R:GTGACGGTGATGCGTACGTT | 194 |
| *mecA* | F: AGTAGAAATGACTGAACGTCCGATAAA  R: GTGCTTTGGTCTTTCTGCATTCCT | 246 |
| *mecC* | F: TTGAGACCAGACGTAATAGTACCT  R: GTGGGACAATACCGATTTCATATGT | 146 |
| *vanA* | F: TACGCAATTGAATCGGCAAGAC  R: GTATCCACACGGGCTAGACCT | 304 |
| *IC* | Same as above | 315 |

**Supplementary file 2: Figure S1**


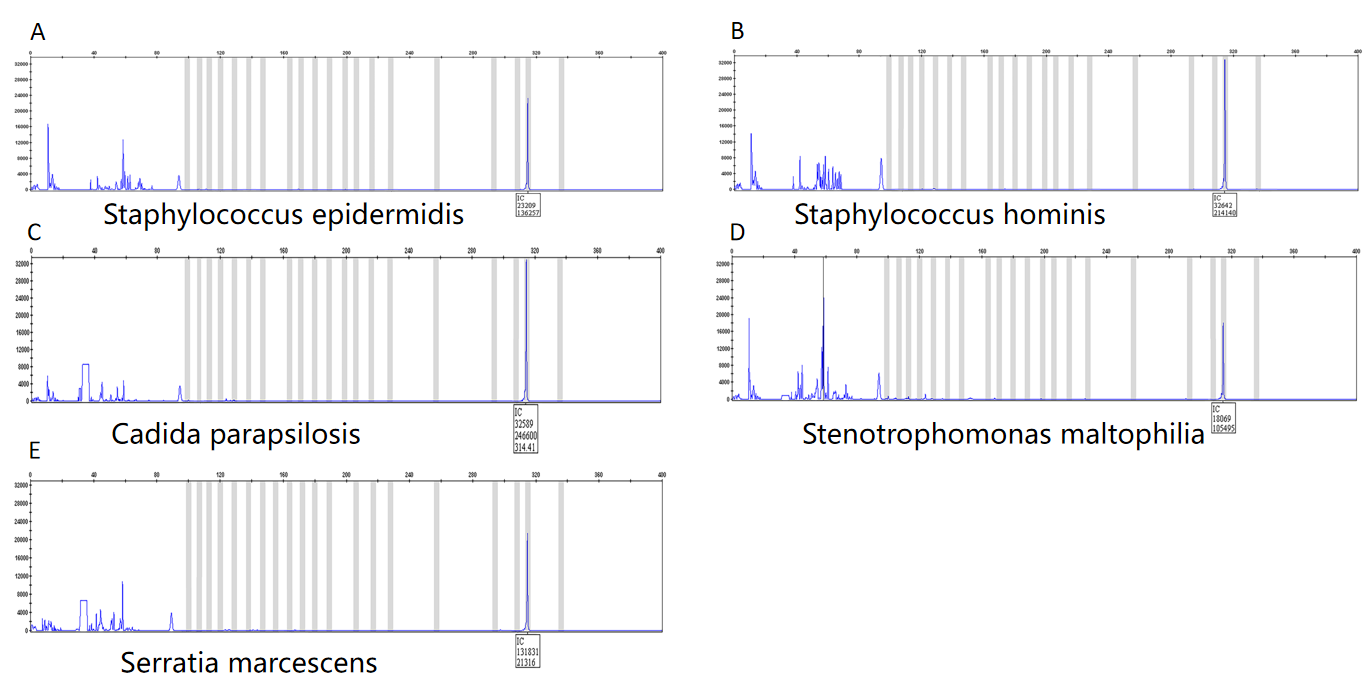


**Supplementary file 2: Figure S1** The detection results of UTI-HMGS of DNA templates from five pathogens for which specific primers were not included in our UTI-HMGS. The result indicated that the uropthogens panel of UTI-HMGS assay did not produce any corresponding specific amplification peaks, similar to the result of negative control ddH2O. (A)The detection result of Staphylococcus epidermidis by UTI-HMGS. (B) The detection result of Staphylococcus hominis by UTI-HMGS. (C) The detection result of Candida parapsilosis by UTI-HMGS. (D) The detection result of Stenotrophomonas maltophilia by UTI-HMGS. (E) The detection result of Serratia marcescens by UTI-HMGS.

**Supplementary file 3: Table S2**

The results of species-level identification by MALDI-TOF and corresponding detection results by UTI-HMGS for 25 urine samples with cocultures

| No | Species of cocultures from MALDI-TOF | detection results by UTI-HMGS | |
| --- | --- | --- | --- |
| 4 | *E. coli* and *E. faecium* | *E. coli* and *E. faecium* | |
| 2 | *E. coli* and *E. faecalis* | *E. coli* and *E. faecalis* |  |
| 2 | *E. coli* and *E. faecalis* | *E. coli*, *E. faecalis* and *K. pneumonia* |  |
| 1 | *E. coli* and *E. faecalis* | *E. coli*, *E. faecalis* and *E. cloacae^#^* |  |
| 1 | *E. coli* and *E. faecalis* | *E. coli*, *E. faecalis*, *P. aeruginosa* and *P. mirabilis* |  |
| 2 | *C. albicans* and *E. faecalis* | *C. albicans* and *E. faecalis* |  |
| 1 | *C. albicans* and *E. faecalis* | *C. albicans*, *E. faecalis*, *P. aeruginosa* and *E. coli* |  |
| 3 | *P. aeruginosa* and *E. faecalis* | *P. aeruginosa* and *E. faecalis* |  |
| 3 | *C. glabrate* and *E. faecium* | *C. glabrate* and *E. faecium* |  |
| 1 | *C. glabrate* and *K. pneumonia* | *C. glabrate* and *K. pneumonia* |  |
| 1 | *P. aeruginosa* and *K. pneumonia* | *P. aeruginosa*, *K. pneumonia* and *E. cloacae* |  |
| 1 | *E. faecalis* and *K. pneumonia* | *E. faecalis*, *K. pneumonia* and *P. aeruginosa* |  |
| 1 | *E. coli* and *P. mirabilis* | *E. coli* and *P. mirabilis* |  |
| 1 | *S. agalactiae* and *E. faecalis* | *S. agalactiae* and *E. faecalis* |  |
| 1 | *C. albicans* and *E. faecium* | *C. albicans*, *E. faecium* and *E. faecalis^#^* |  |

# indicate the detection peak area of pathogen by UTI-HMGS is below the corresponding semi-quantitative cut-off values cut-off value and culture negative. There were 4 urine samples with cocultures were detected one pathogen by UTI-HMGS with the detection peak area were all above cut-off values but culture negative; similarly, 2 urine sample were detected two different pathogens.

**Supplementary file 4: Table S3**

The primer sequence of each pathogen for conventional PCR and product sizes for sanger sequencing

| targets of dection | sequences (5′→3′) |
| --- | --- |
| Uropathogens |  |
| *E. coli* | F:CGCAAATGGGCGGTAGGCGTGCTCAGCGGTCGTGGTAGATG |
|  | R: GTTCACGGTGCCCTCCCCGTACTGCCGTCTAGAATCA |
| *K. pneumonia* | F:CGCAAATGGGCGGTAGGCGTGGCTTATACCGGCATTCCATTGTA  R: GTTCACGGTGCCCTCCCGACCGTACCGCAGATATCA |
| *P. mirabilis* | F:CGCAAATGGGCGGTAGGCGTGCAGATCCTTGCAGAGCTACAG  R: GTTCACGGTGCCCTCCTCTCTTAACTGACGATAAGCCACAT |
| *P. aeruginosa* | F:CGCAAATGGGCGGTAGGCGTGCGGCTACTTCTTCACCATCCACT  R: GTTCACGGTGCCCTCCAAGCAGTGTTCAACCGGACCT |
| *A. baumannii* | F:CGCAAATGGGCGGTAGGCGTGTGGTGAGCTTCGCGTATATGTT  R: GTTCACGGTGCCCTCCGCACCGATTAAACCGCCAAG |
| *E. cloacae* | F:CGCAAATGGGCGGTAGGCGTGCGTTTAACCCGGTAGATGAGA  R: GTTCACGGTGCCCTCCGGTCAGCTGTTTGTTGGAGA |
| *E .faecalis* | F:CGCAAATGGGCGGTAGGCGTGGCGAAATTGGGAAGGCTGTTG |
|  | R: GTTCACGGTGCCCTCCAGGTTGCTTCATCTTCATCGGT |
| *E. faecium* | F:CGCAAATGGGCGGTAGGCGTGGGACTGATCATTGGTGCTGT  R: GTTCACGGTGCCCTCCTCAGCAGCCGTAACTACACC |
| *S. aureus* | F:CGCAAATGGGCGGTAGGCGTGCAGAGTTACATGTTTCGGATGCT  R: GTTCACGGTGCCCTCCCACCTGCAGCATCCTTACCT |
| *S. agalactiae* | F:CGCAAATGGGCGGTAGGCGTGTGACTAGTGAGGATGCCGTT  R: GTTCACGGTGCCCTCCGCTCGTGTCAATTCAGGCAA |
| *C. albicans* | F:CGCAAATGGGCGGTAGGCGTGTGAGTTCGGTTGGGACTAGC |
|  | R: GTTCACGGTGCCCTCCGCGCTACTTTGCATAACCGT |
| *C. glabrata* | F:CGCAAATGGGCGGTAGGCGTGAGGACGCCATATCAAGAGGA  R: GTTCACGGTGCCCTCCTGACTTATCATCTACAGTGCTTCCA |
| *C. tropicalis* | F:CGCAAATGGGCGGTAGGCGTGTGTCATCTCAACGACCCAAACT |
|  | R: GTTCACGGTGCCCTCCCAAATCAGCTGGCATGGCAA |
| *CT* | F:CGCAAATGGGCGGTAGGCGTGAGAAGACACAAACCCGCACA  R: GTTCACGGTGCCCTCCAAAGGGCCCAGGTTGATTGT |
| *UU* | F:CGCAAATGGGCGGTAGGCGTGAGATGCTGCTACAACTCCAGA  R: GTTCACGGTGCCCTCCTCATACCTAATTGTACTGTACCCTCA |
| *MH* | F:CGCAAATGGGCGGTAGGCGTGTTTATTGGGCTCGGTCACAG  R: GTTCACGGTGCCCTCCTGCATATCTTCAGAGTTACCTATTGTA |
| *UTB* | F:CGCAAATGGGCGGTAGGCGTGTCCACTGATTACGGCCAACA  R: GTTCACGGTGCCCTCCGTCGACGACTTGTAGCGCTT |
| *NG* | F:CGCAAATGGGCGGTAGGCGTGAGCCGTACCTTCAACCTGATG  R: GTTCACGGTGCCCTCCGACCAAACCGATCAGAGAGGA |
| resistance genes |  |
| *CTX-M* | F:CGTCACGCTGTTGTTAGGAA  R:GAATGGCGGTGTTTAACGTC |
| *SHV*  *KPC* | F:TTGCGTTATATTCGCCTGTGTATTATCTC  R:GTGCTGGCGATAGTGGATCTTTC  F:GTCTAGTTCTGCTGTCTTGTCT  R:GGGATGGCGGAGTTCAG |
| *NDM* | F:CCAATATTATGCACCCGGTC  R:CGACAACGCATTGGCAT |
| *IMP* | F:CTTTATATTTTTGTTTTGCAGCATTG  R:TTCAGGCAACCAAACCAC |
| *VIM* | F:ATCATGGCTATTGCGAGTCC  R:ACTCATAAATCGCACAACCAC |
| *mecA* | F:GGGTTTGGTATATATTTTTATGCTTC  R:AAATACTTAGTTCTTTAGCGATTG |
| *mecC* | F:TCTAAACTGGCATATGGAGAAGA  R:TCGTTCAATGGATAAACACGG |
| *vanA* | F:TCTGTTTGAATTGTCCGGTA  R:CCCCTTTAACGCTAATACGAT |

**Supplementary file 5:** **Table S4**

For 266 clinical urine sample with monomicrobial cultures, the UTI-HMGS detected pathogens with the peak area above or far below the corresponding semi-quantitative cut-off values but had no cultures

| No | Culture positive for organism | Detection results of UTI-HMGS |
| --- | --- | --- |
| 2 | *E. coli* | *E. coli**and *E. faecalis* |
| 1 | *E. coli* | *E. coli**and *P. aeruginosa* |
| 2 | *E. coli* | *E. coli**and *MH* |
| 1 | *E. coli* | *E. coli**, *P. aeruginosa* and *E. faecalis* |
| 1 | *E. coli* | *E. coli**, *K. pneumonia* and *MH* |
| 1 | *E. coli* | *E. coli**and *UU* |
| 1 | *E. coli* | *E. coli**and *NG* |
| 2 | *K. pneumonia* | *K. pneumonia**and *C. glabrate* |
| 2 | *K. pneumonia* | *K. pneumonia**and *C. albicans** |
| 1 | *K. pneumonia* | *K. pneumonia**and *E. faecalis* |
| 1 | *K. pneumonia* | *K. pneumonia**and *UU* |
| 1 | *K. pneumonia* | *K. pneumonia**, *E. faecalis*, *E. faecium and E. coli* |
| 1 | *K. pneumonia* | *K. pneumonia**and *MH* |
| 4 | *C. albicans* | *C. albicans** and *E. faecium* |
| 2 | *C. albicans* | *C. albicans** and *E. coli* |
| 1 | *C. albicans* | *C. albicans**, *K. pneumonia* and *C. glabrate** |
| 3 | *C. tropicalis* | *C. tropicalis** and *E. faecium* |
| 1 | *C. tropicalis* | *C. tropicalis**, *E. faecium** and *K. pneumonia* |
| 1 | *C. tropicalis* | *C. tropicalis**, *C. glabrate**, *E. coli** and *E. faecium** |
| 1 | *P. mirabilis* | *P. mirabilis**and *E. faecalis* |
| 1 | *P. mirabilis* | *P. mirabilis**and *K. pneumonia* |
| 1 | *P. mirabilis* | *P. mirabilis**, *E. faecium* and *E. faecalis* |
| 1 | *E. cloacae* | *E. cloacae** and *E. faecium* |
| 1 | *E. cloacae* | *E. cloacae** and *E. faecalis* |
| 1 | *E. cloacae* | *E. cloacae** and *S. aureus* |
| 2 | *A. baumannii* | *A. baumannii**and *E. faecalis* |
| *1* | *A. baumannii* | *A. baumannii**, *P. mirabilis** and *K. pneumonia* |
| *1* | *P. aeruginosa* | *P. aeruginosa**and *E. faecalis** |
| *1* | *P. aeruginosa* | *P. aeruginosa**and *E. faecalis* |
| 1 | *P. aeruginosa* | *P. aeruginosa**and *E. coli* |
| 1 | *P. aeruginosa* | *P. aeruginosa**and *C. albicans* |
| 1 | *P. aeruginosa* | *P. aeruginosa**, *E. faecium** and *E. faecalis** |
| 1 | *P. aeruginosa* | *P. aeruginosa**, *E. faecium* and *C. albicans* |
| 1 | *P. aeruginosa* | *P. aeruginosa**, *E. faecalis* and *E. cloacae* |
| 1 | *P. aeruginosa* | *P. aeruginosa**, *E. coli* and *K. pneumonia* |
| 1 | *P. aeruginosa* | *P. aeruginosa**, *E. faecalis*, *P. mirabilis*, *S. agalactiae* and *E. coli* |
| 3 | *E. faecium* | *E. faecium**and *C. glabrate** |
| *1* | *E. faecium* | *E. faecium**and *C. glabrate* |
| *3* | *E. faecium* | *E. faecium**and *C. albicans* |
| *2* | *E. faecium* | *E. faecium** and *E. coli* |
| *2* | *E. faecium* | *E. faecium** and *K. pneumonia* |
| *1* | *E. faecium* | *E. faecium**, *K. pneumonia** and *E. coli* |
| *6* | *E. faecalis* | *E. faecalis**and *C. albicans* |
| *2* | *E. faecalis* | *E. faecalis**, *E. faecium** and *K. pneumonia* |
| *1* | *E. faecalis* | *E. faecalis** and *K. pneumonia* |
| *1* | *E. faecalis* | *E. faecalis** and *E. coli* |
| *1* | *E. faecalis* | *E. faecalis**, *E. coli* and *E. cloacae* |
| *1* | *E. faecalis* | *E. faecalis** and *S. agalactiae* |
| *1* | *E. faecalis* | *E. faecalis** and NG |
| *1* | *E. faecalis* | *E. faecalis**, *C. albicans*, *E. coli* and NG |
| *2* | *S. aureus* | *S. aureus**and *E. faecalis* |
| *1* | *S. aureus* | *S. aureus**and *E. faecalis** |
| *2* | *S. aureus* | *S. aureus**and *E. faecium* |
| *1* | *S. agalactiae* | *S. agalactiae**and *UU* |
| *1* | *S. agalactiae* | *S. agalactiae**and *NG* |

* indicate the detection peak area of pathogen by UTI-HMGS is above the corresponding semi-quantitative cut-off values cut-off value and culture negative. There were 15 urine samples with monomicrobial culture were detected one pathogen by UTI-HMGS with the detection peak area were all above cut-off values but culture negative; similarly, 1 urine sample were detected two different pathogens and 1 urine sample were detected three different pathogens.

There were 53 urine samples with monomicrobial culture were detected one pathogen by UTI-HMGS with the detection peak area were all far below cut-off values and culture negative; similarly, 7 urine samples were detected two different pathogens;1 urine sample were detected three different pathogens and 1 urine sample were detected four different pathogens.

Additionally, 11 urine samples were detected one different difficult-to-culture pathogen, include 4*NG*, 4*MH* and 3*UU*.

**Supplementary file 6: Table S5**

For 240 clinical urine samples with negative urine culture, the UTI-HMGS detected pathogens with the peak area above or far below the corresponding semi-quantitative cut-off values cut-off values but had no cultures

| No | Detection results of UTI-HMGS |
| --- | --- |
| 15 | *UU* |
| 1 | *UU* and *P. aeruginosa* |
| 1 | *UU* and *P. aeruginosa* |
| 1 | *UU* and *E. coli* |
| 1 | *UU*, *MH* and *E. coli* |
| 1 | *UU*, *MH*, *K. pneumonia*, *C. albicans* and *E. coli* |
| 4 | *CT* |
| 1 | *MH* |
| 1 | *NG* |
| 1 | *NG* and *E. coli* |
| 2 | *UTB* |
| 1 | *UTB* and *K. pneumonia** |
| 3 | *E. faecalis** |
| 2 | *K. pneumonia** |
| 1 | *E. coli** |
| 1 | *C. albicans** |
| 1 | *E. faecalis** and *E. coli** |
| 1 | *E. faecium** and *K. pneumonia* |
| 1 | *C. albicans**, *K. pneumonia* and *A. baumannii* |
| 1 | *E. faecalis**, *P. aeruginosa**, *C. albicans* and *E. coli* |
| 1 | *E. cloacae** |
| 13 | *E. coli* |
| 8 | *E. faecium* |
| 7 | *K. pneumonia* |
| 7 | *E. faecalis* |
| 4 | *P. aeruginosa* |
| 3 | *C. albicans* |
| 3 | *A. baumannii* |
| 2 | *P. mirabilis* |
| 1 | *P. mirabilis* |
| 1 | *S. aureus* |
| 1 | *S. agalactiae* |
| 1 | *E. cloacae* |
| 2 | *C. albicans* and *E. coli* |
| 2 | *K. pneumonia* and *C. albicans* |
| 2 | *K. pneumonia* and *E. coli* |
| 2 | *E. faecalis* and *C. albicans* |
| 2 | *E. faecalis* and *E. coli* |
| 1 | *C. albicans* and *P. mirabilis* |
| 1 | *C. albicans* and *C. tropicalis* |
| 1 | *A. baumannii* and *C. albicans* |
| 1 | *A. baumannii* and *E. coli* |
| 1 | *E. faecalis* and *S. aureus* |
| 1 | *E. faecalis* and *P. mirabilis* |
| 1 | *E. faecalis* and *E. faecium* |
| 1 | *E. faecalis* and *P. aeruginosa* |
| 1 | *E. faecalis* and *S. agalactiae* |
| 1 | *E. faecium* and *E. coli* |
| 1 | *P. aeruginosa* and *E. coli* |
| 1 | *P. aeruginosa* and *C. tropicalis* |
| 1 | *P. aeruginosa* and *S. agalactiae* |
| 1 | *S. agalactiae* and *E. coli* |
| 1 | *E. cloacae* and *E. coli* |
| 1 | *E. faecalis*, *A. baumannii* and *C. albicans* |
| 1 | *E. faecalis*, *E. coli* and *K. pneumonia* |
| 1 | *E. faecalis*, *E. coli* and *P. mirabilis* |
| 1 | *P. aeruginosa*, *E. faecium* and *E. faecalis* |
| 1 | *P. aeruginosa*, *E. faecium* and *C. albicans* |
| 1 | *P. aeruginosa*, *C. tropicalis* and *E. coli* |

* indicate the detection peak area of pathogen by UTI-HMGS is above the corresponding semi-quantitative cut-off values cut-off value and culture negative. For 240 clinical urine samples with negative urine culture, 13were detected 1-2 pathogens with the peak area above the corresponding semi-quantitative cut-off values (11 were detected one pathogen and 2 were detected two different pathogens); 58 were detected one pathogen by UTI-HMGS with the detection peak area were all far below cut-off values and culture; similarly, 27 urine samples were detected two different pathogens and 7 urine sample were detected three different pathogens. Additionally, 30 urine samples were detected four different difficult-to-culture pathogens, include 18*UU*, 4*CT*, 3*UTB*, 2*NG*, 1*MH* and 2*UU*+*MH.*
